# SEEDSTICK is a Master Regulator of Development and Metabolism in the Arabidopsis Seed Coat

**DOI:** 10.1371/journal.pgen.1004856

**Published:** 2014-12-18

**Authors:** Chiara Mizzotti, Ignacio Ezquer, Dario Paolo, Paloma Rueda-Romero, Rosalinda Fiorella Guerra, Raffaella Battaglia, Ilana Rogachev, Asaph Aharoni, Martin M. Kater, Elisabetta Caporali, Lucia Colombo

**Affiliations:** 1 Dipartimento di BioScienze, Università degli Studi di Milano, Milan, Italy; 2 Consiglio Nazionale delle Ricerche, Istituto di Biofisica, Milan, Italy; 3 Centro de Biotecnología y Genómica de Plantas-UPM-INIA, ETSI Agrónomos, Universidad Politécnica de Madrid, Campus de Montegancedo, Madrid, Spain; 4 Department of Plant Sciences, Weizmann Institute of Science, Rehovot, Israel; North Carolina State University, United States of America

## Abstract

The role of secondary metabolites in the determination of cell identity has been an area of particular interest over recent years, and studies strongly indicate a connection between cell fate and the regulation of enzymes involved in secondary metabolism. In *Arabidopsis thaliana*, the maternally derived seed coat plays pivotal roles in both the protection of the developing embryo and the first steps of germination. In this regard, a characteristic feature of seed coat development is the accumulation of proanthocyanidins (PAs - a class of phenylpropanoid metabolites) in the innermost layer of the seed coat. Our genome-wide transcriptomic analysis suggests that the ovule identity factor SEEDSTICK (STK) is involved in the regulation of several metabolic processes, providing a strong basis for a connection between cell fate determination, development and metabolism. Using phenotypic, genetic, biochemical and transcriptomic approaches, we have focused specifically on the role of *STK* in PA biosynthesis. Our results indicate that STK exerts its effect by direct regulation of the gene encoding *BANYULS*/*ANTHOCYANIDIN REDUCTASE* (*BAN*/*ANR*), which converts anthocyanidins into their corresponding 2,3-cis-flavan-3-ols. Our study also demonstrates that the levels of H3K9ac chromatin modification directly correlate with the active state of *BAN* in an STK-dependent way. This is consistent with the idea that MADS-domain proteins control the expression of their target genes through the modification of chromatin states. STK might thus recruit or regulate histone modifying factors to control their activity. In addition, we show that STK is able to regulate other *BAN* regulators. Our study demonstrates for the first time how a floral homeotic gene controls tissue identity through the regulation of a wide range of processes including the accumulation of secondary metabolites.

## Author Summary

Plant secondary metabolites accumulate in seeds to protect the developing embryo. Using an RNA sequencing approach in conjunction with enrichment analyses we identified the homeotic MADS-domain gene *SEEDSTICK* (*STK*) as a regulator of metabolic processes during seed development. We analyzed the role of *STK* as a key regulator of the production of proanthocyanidins, compounds which are important for the pigmentation of the seed. STK directly regulates a network of metabolic genes, and is also implicated in changes occurring in the chromatin landscape. Our work demonstrates that a key homeotic transcription factor not only determines the identity of ovules but also controls metabolic processes that occur subsequent to the initial identity determination process, thus suggesting a link between identity determination and cell-specific (metabolic) processes.

## Introduction

Seeds are essential units for plant propagation. Their development is an intricate process that requires the coordinated development of the embryo, the endosperm and the seed coat. The seed coat is derived from the maternal integuments and surrounds the embryo providing the latter protection against both mechanical damage as well as that inflicted by UV radiation. In addition, it facilitates the efficient dispersion of offspring and mediates initial water uptake during germination [1]. Upon double fertilization, the first phase of seed development is characterized by several morphological changes followed by the accumulation of secondary metabolites in specialized seed coat cells which mainly act in defence responses [2]. Although some interconnections between secondary metabolism and cell differentiation have been reported, the molecular mechanisms involved have not yet been elucidated. New approaches to genome-wide target identification indicate the existence of a correlation between cell identity specification and the regulation of secondary metabolism. For example, direct targets of the MADS-domain transcription factor SEPALLATA3 (SEP3) have been shown to include genes involved in lipid biosynthesis, hormone production and the biosynthesis of sterol and wax [3]; SHORT VEGETATIVE PHASE (SVP), another MADS-domain protein, binds to genes involved in the hormone stimulus response, suggesting its involvement in the cytokinin, auxin and jasmonate signalling pathways [4]. MADS-domain transcription factors have been demonstrated to be important regulators of floral organ specification, and SEEDSTICK (STK) in particular has been shown to play a pivotal role in ovule ontogeny [5]. The *STK* gene controls ovule identity redundantly with *SHATTERPROOF1* (*SHP1*) and *SHP2* [6]–[8]. Furthermore, *STK* is required for normal seed shedding and, together with another MADS-domain gene *ARABIDOPSIS B SISTER* (*ABS*), for proper formation of the endothelium, the innermost layer of the seed coat [7], [9]. Transcriptome analysis of developing ovules and seeds has suggested the involvement of STK in the control of secondary metabolism (see later). Based on the data obtained and the morphological characterization of the *stk* mutant we have focused on the regulation of the flavonoid metabolic pathway.

Fertilized ovules accumulate proanthocyanidins (PAs) in the endothelium. These molecules are flavan-3-ols and in Arabidopsis they are composed of epicatechin monomers and polymers [10]. PAs are important compounds as they provide protection against light and predation by herbivores, they have antimicrobial and antioxidant activities, and in addition they limit the growth of neighbouring plants [11]–[16]. Epicatechins are responsible for the brown pigmentation of the Arabidopsis seed [16]. They are synthesized in the cytoplasm and then transported to the vacuoles where finally they are polymerized [17]. In Arabidopsis, PA biosynthesis begins in the micropylar region of the endothelium around 1 to 2 days after fertilization and then progressively extends to include the rest of the endothelium up to 5 to 6 days after fertilization [2], [16]. The genetics of PA biosynthesis and accumulation has been well-studied in Arabidopsis revealing the participation of a complex network of several groups of genes. PA biosynthetic structural genes are divided into two groups: the Early (EBGs) and the Late (LBGs) Biosynthetic Genes [16], [18], [19]. The EBGs comprise four genes: *CHALCONE SYNTHASE* (*CHS*), *CHALCONE ISOMERASE* (*CHI*), *FLAVONOL 3-HYDROXYLASE* (*F3H*) and *FLAVONOL 3′-HYDROXYLASE* (*F3′H*), and are involved in the biosynthesis of precursors for PAs and other classes of Arabidopsis flavonoids. The LBGs include *DIHYDROFLAVONOL-4-REDUCTASE* (*DFR*), *LEUCOANTHOCYANIDIN DIOXYGENASE* (*LDOX*) and *BANYULS*/*ANTHOCYANIDIN REDUCTASE* (*BAN*/*ANR*). *BAN* is considered to be a branch point in the phenylpropanoid biosynthetic pathway. It encodes an anthocyanidin reductase which converts anthocyanidins to their corresponding 2,3-cis-flavan-3-ols [20]. A third group of structural genes has also been identified in Arabidopsis that comprises *TRANSPARENT TESTA 12* (*TT12*, *MATE transporter*), *TT10* (*laccase 15*), *TT19* (*glutathione-S-transferase*) and *AHA10* (*H^+^-ATPase*). These genes are involved in flavan-3-ol modification, transport and oxidation [21]–[24] and have recently been proposed to be LBG members [19]. The regulation of LBGs occurs via a ternary protein complex called MBW (MYB-bHLH-WDR), formed by a specific R2R3-MYB, a bHLH transcription factor and the WD repeat protein TRANSPARENT TESTA GLABRA 1 (TTG1; [16], [25], [26]). In addition to this complex, other transcription factors belonging to different families such as Zinc finger (*TT1*/*WIP1*), MADS (*ABS*/*TT16*/*AGL32*) and WRKY (*TTG2*/*DSL1*/*WRKY44*) also participate in their regulation [16], [27]–[29].

Studies in several fields indicate a correlation between cell identity determination and metabolism. In unicellular organisms, like some species of yeast and *Streptomyces*, cellular differentiation is influenced by nutrients and metabolism [30], [31]. Some years ago it was proposed that enzymes involved in carbon metabolism also regulate myoblast differentiation [32], [33], while stem cell differentiation has recently been proposed to be regulated by the metabolisms of both lipids and methionine [34], [35].

The results presented in this manuscript demonstrate that STK directly prevents ectopic accumulation of PAs in the seed coat. STK both directly and indirectly regulates *BAN* and this involves STK-dependent changes in histone modification. The discovery of STK as a master regulator of genes involved in the anthocyanin biosynthetic pathway provides an interesting link between the determination of organ identity and more downstream cell-specific metabolic processes. Furthermore it opens up new possibilities to increase the levels of bioactive natural products which constitute a rich source of novel therapeutic compounds, or to modify pigments in plant tissues.

## Results

### *STK* is involved in the regulation of metabolic pathways

**Figure 1:**
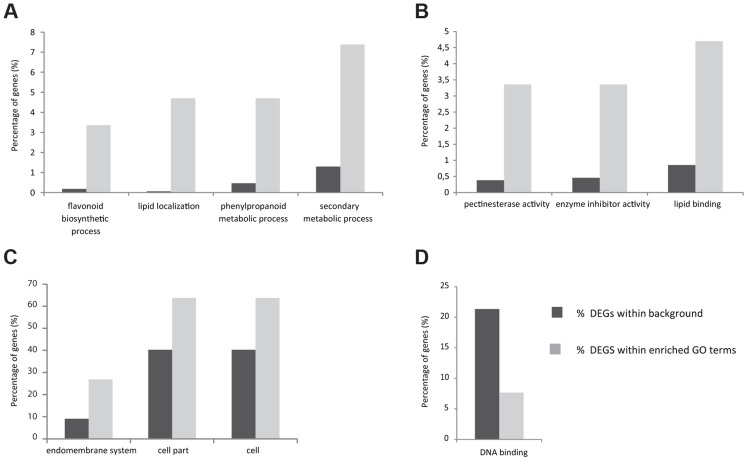
Histogram of functional gene ontology analysis of differentially expressed genes. Slim Plant term enrichment - up and down-regulated genes. Genes with higher expression in *stk* in the Biological Process (*A*), Molecular Function (*B*) and Cellular Component (*C*) categories. Genes with lower expression in *stk* Molecular Function category (*D*).

As a first step to understand which processes are controlled by STK, a high-throughput RNA-Seq analysis was performed comparing wild-type plants with the *stk* mutant. Based on the *STK* expression pattern, RNA was extracted from flowers starting from early stages of development (stage 9) until maturity and after fertilization until 5 Days After Pollination (DAP). Analysis of the raw data was performed on the commercially available CLC Genomics Workbench v.4.7.1 (http://www.clcbio.com/genomics/). A total of 102,278,242 reads passed a quality filter and 85% were mapped back to the Arabidopsis TAIR10 genome. Approximately 90% of these mapped uniquely to single locations and each could thus be assigned to a single annotated TAIR10 gene. Normalization of expression was performed using RPKM values [36]. All other parameters were kept at default levels. The CLC Genomic Workbench was further used to identify and assess the levels of all the differentially expressed transcripts found in each cDNA library. Baggerley's test and False Discovery Rate (FDR) correction were used for the statistical evaluation of samples [37]. Our analysis revealed that 156 genes were up-regulated (S1 Table) in the *stk* mutant compared to wild type, whereas 90 were found to be down-regulated (S2 Table). To obtain an initial insight into the potential functions of *STK* downstream genes, a global view of function and the underlying biology of the differentially expressed genes was obtained by examining their gene ontology using agriGO (Fig. 1; [38]). For up-regulated genes in the *stk* mutant, the biological process category showed enrichment for lipid localization and secondary metabolic processes (Fig. 1A; S3 Table). There was also notable enrichment for terms related to the phenylpropanoid metabolic process as well as flavonoid biosynthesis. Analysis of the molecular functions affected revealed enrichment for genes encoding pectinesterase, enzyme inhibitor and lipid binding activities (Fig. 1B; S3 Table). Analysis of enriched cellular component terms in the list of up-regulated genes included categories related to the endomembrane system and also cell and cell part groups (Fig. 1C; S3 Table). Among the group of genes down-regulated in *stk*, we only found significant enrichment of terms in the molecular function category related to DNA binding (Fig. 1D; S3 Table). The transcriptome picture emerging provided a global view of the downstream networks regulated by STK. Interestingly, we found genes involved in flavonoid biosynthesis to be significantly represented in the up-regulated genes. That this group included key enzymatic players involved in PA synthesis constituted the basis for the work we report here on deciphering the role of STK in this seed coat process.

### The *stk* mutant accumulates PAs ectopically

**Figure 2:**
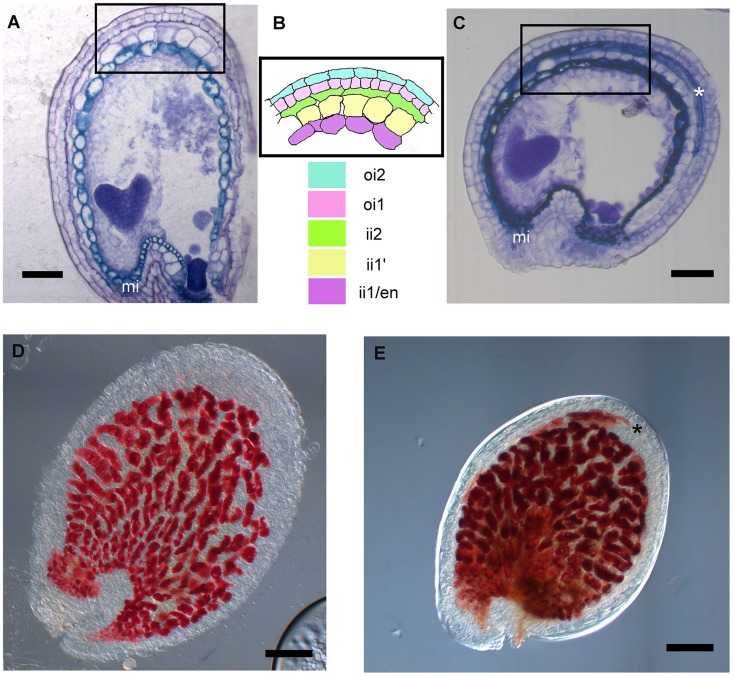
*stk* mutant seeds present defects in seed coat PA accumulation. (*A*) Sections of wild-type seeds stained with the toluidine blue O revealed the presence of phenolic compounds in the endothelium (ii1). (*B*) Scheme of Arabidopsis seed coat anatomy. (*C*) In the *stk* mutant phenolic compounds are accumulated in the endothelium (ii1) and also in the second layer of the inner integument (ii2, asterisk). (*D*) Whole-mount vanillin staining confirmed the presence of PAs in the wild-type and (*E*) in the *stk* mutant endothelium. In the *stk* mutant PAs are also accumulated outside the endothelium in the second layer of the inner integument (asterisk). mi, micropyle; en, endothelium. Scale bars = 30 µm (*A–E*).

The transcriptomic analysis of *stk* mutant ovules and seeds highlighted a significant increase in the abundance of transcripts involved in secondary metabolic processes, including those of genes involved in flavonoid and phenylpropanoid biosynthesis. Interestingly, in this group we found *DFR*, *LDOX* and *BAN*, three key enzyme encoding genes controlling the synthesis of catechin and epicatechin, the precursors of PAs. We therefore decided to study the role of STK in the regulation of the PA biosynthetic pathway in more detail since this is considered to be a key metabolic pathway linked to seed development (for review see [16]). Furthermore, recent discoveries have shown that flavonoids play a fundamental role in regulating communication between the seed coat and the endosperm [39]. PAs in wild-type Arabidopsis seeds are accumulated in the endothelium. To investigate and compare the accumulation of PAs in wild-type and *stk* mutant seeds we made seed sections at the heart stage of embryo development (when PA accumulation in the endothelium is completed) and stained these with toluidine blue O. This staining provided a general view of all seed coat cells and revealed the presence of phenolic compounds in the endothelium of wild-type and *stk* mutant seeds as highlighted by the blue staining of their vacuoles (Fig. 2A–C). However, the toluidine blue also evidenced considerable accumulation of phenolic compounds in the outermost layer of the inner integument (ii2) in the *stk* mutant seed coat (Fig. 2C, asterisk). In order to confirm the nature of the latter we used the vanillin assay that specifically detects flavan-3-ols and their proanthocyanidin polymers [2]. This analysis confirmed that PAs are accumulated in the endothelium in wild-type seeds (Fig. 2D) whereas in the *stk* mutant they are additionally observed in the ii2 layer (Fig. 2E, asterisk).

**Figure 3:**
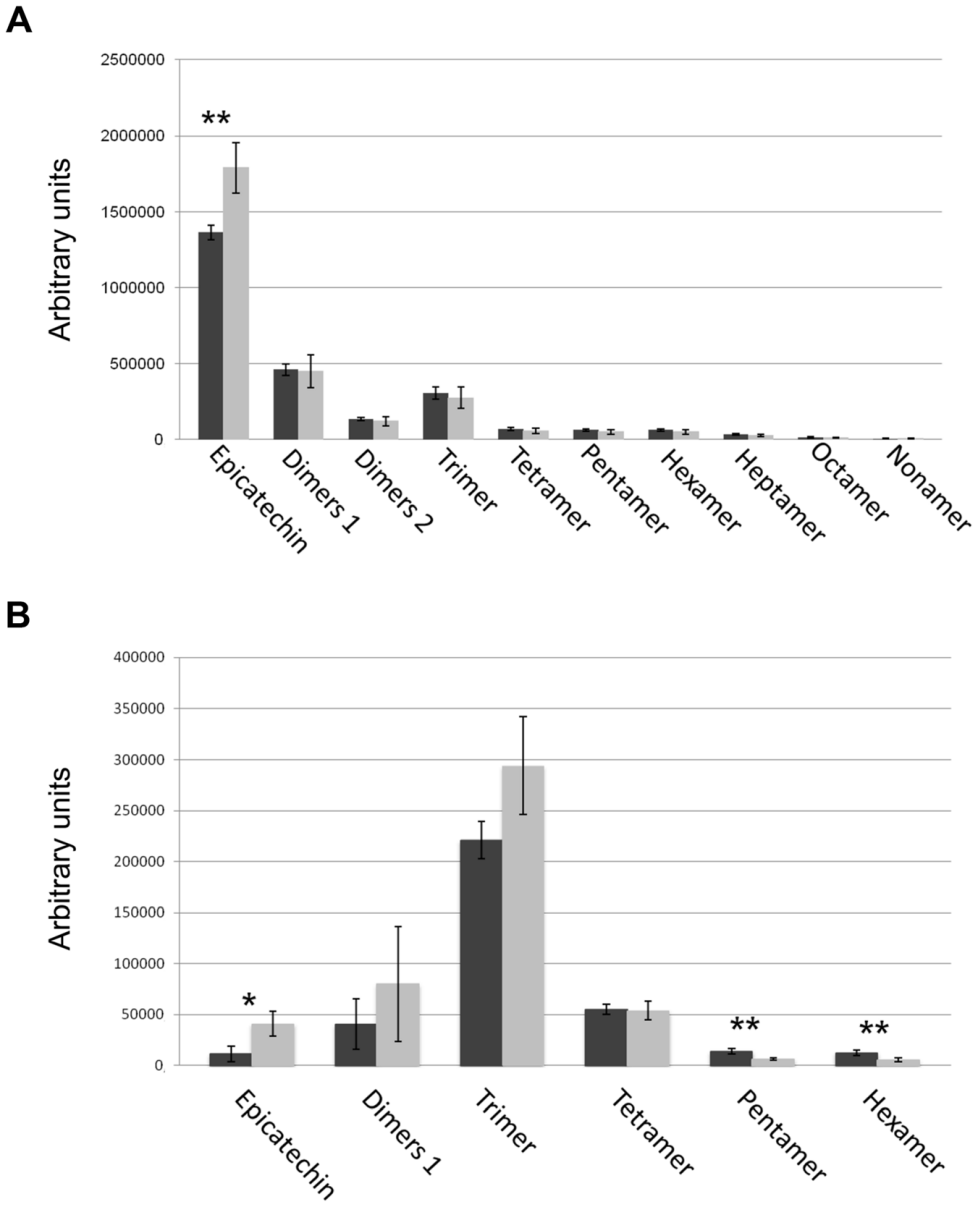
Solvent soluble PAs analysed by LC-MS. Soluble PAs were detected in wild type (black bars) and the *stk* mutant (grey bars) at the immature (6 DAP; *A*) and mature (*B*) stages of seed development. Error bars represent SD of three independent measurements. Asterisks indicate statistically significant differences as determined by Student's *t* test (* *P*<0.05, ** *P*<0.01).

To better understand the role of STK in PA synthesis, soluble and insoluble extracts from mature and immature (6 DAP) seeds were analyzed by Liquid Chromatography-Mass Spectrometry (LC-MS) and the complete metabolic profiles obtained are shown in Fig. 3. Peaks that could be identified as known compounds were selected and analyzed (S4 Table). No differences were observed in the metabolic profiles of insoluble PAs between mature wild-type and *stk* seeds (S1 Figure). At 6 DAP, insoluble PAs could not be detected which concords with the solvent-soluble nature of PAs at the immature stage [10]. Soluble PAs, however, showed some differences: at 6 DAP, both wild-type and *stk* mutant seeds contained the same levels of PA oligomers (n = 2–9) but the level of epicatechin monomers was higher in the *stk* mutant compared to the wild type (Fig. 3A). In mature seeds only six soluble PA metabolites were detected. The levels of dimers, trimers and tetramers were the same in the wild type and in the mutant, but the levels of epicatechin monomers, pentamers and hexamers were different (Fig. 3B). In particular, the levels of pentamers and hexamers in the wild type were higher compared to the mutant. By contrast the level of epicatechin monomers was higher in the *stk* mutant, as already detected in the metabolic profiles of immature seeds. The total amount of PAs in the *stk* mutant was greater than in wild-type seeds. These data support the morphological analysis and suggest that *stk* mutant seeds have a higher level of PAs than wild-type seeds. Furthermore, it implies that STK is predominantly involved in epicatechin monomer metabolism and only slightly affects oligomer production.

### STK negatively controls key regulators of the PA biosynthetic pathway

**Figure 4:**
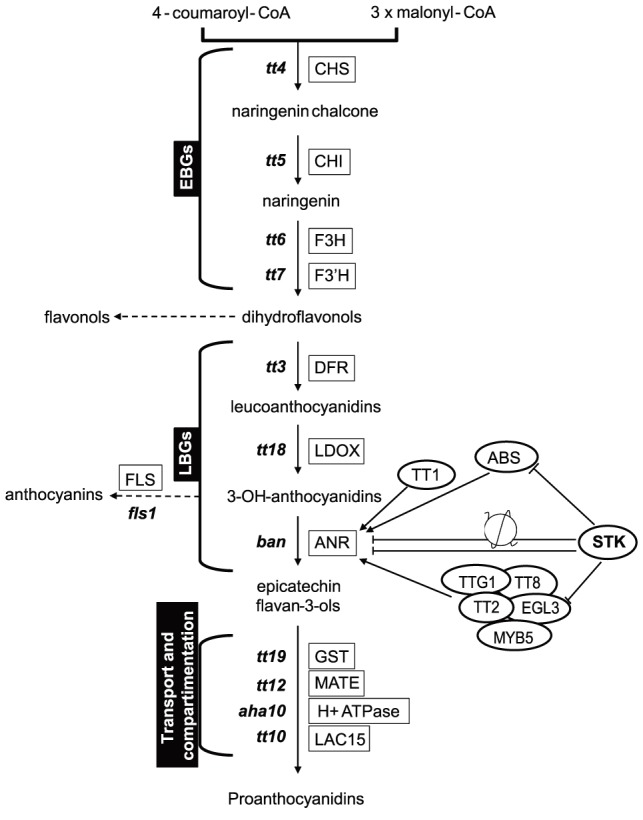
*STK* has a pivotal role in the control of PA production. Schematic representation of the pathways for PA production. The genes involved in these pathways are shown in boxes. *BAN*, that was found to be up-regulated in RNA-Seq, has been analysed by *in situ* hybridization, qRT-PCR and ChIP assay. The ChIP assay demonstrated that STK directly regulates *BAN*, *ABS* and *EGL3* (solid line). The ChIP assay revealed that STK function negatively correlates with the level of the H3K9ac mark on the *BAN* promoter (solid line with nucleosome).

The biosynthesis of PAs is dependent on structural genes that can be divided into EBGs and LBGs (Fig. 4), the EBGs being expressed prior to the LBGs [16]. Based on the transcriptome data, we focused our attention on the set of genes controlling the transformation of anthocyanidin into epicatechin (Fig. 4). The RNA-Seq data revealed that the expression of genes belonging to the EBGs is unaltered in the *stk* mutant background (Table 1); however, the levels of all the LBGs were found to be increased in the mutant (Table 1). This suggests that STK acts as a repressor of the expression of all the LBGs. Among these, *BAN* codes for the core enzyme of PA production [25], [40]–[42]. We performed quantitative Real Time-PCR (qRT-PCR) experiments on siliques from 0 to 6 DAP and confirmed the results obtained by the RNA-Seq experiment showing *BAN* to be up-regulated in the *stk* mutant (S2 Figure).

**Table 1:** Summary of the RNA sequencing results for the genes involved in the PA pathway.

	*Difference*	*Fold Change*
**Early Biosynthetic Genes (EBGs)**		
*CHALCONE SYNTHASE (CHS)*	−40.86	−1.05*
*CHALCONE ISOMERASE (CHI)*	−3.35	−1.04
*FLAVANONE-3-HYDROXYLASE (F3H)*	−5.97	−1.05
*FLAVANONE-3′-HYDROXYLASE (F3′H)*	+2.67	+1.04
*FLAVONOL SYNTHASE (FLS)*	−37.15	−1.18
**Late Biosynthetic Genes (LBGs)**		
*DIHYDROFLAVONOL REDUCTASE (DFR)*	+26.13	+2.2**
*LEUCOCYANIDIN DIOXYGENASE (LDOX)*	+29.71	+2.35**
*ANTHOCYANIDIN REDUCTASE (BAN)*	+45.21	+2.18**
**Regulatory Genes**		
*ARABIDOPSIS Bsister (ABS)*	+0.33	+1.64
*TRANSPARENT TESTA GLABRA 1 (TTG1)*	−0.27	−1.01
*TRANSPARENT TESTA GLABRA 2 (TTG2)*	+0.74	+1.27
*TRANSPARENT TESTA 1 (TT1)*	+1.64	+2.03
*TRANSPARENT TESTA 2 (TT2)*	+3.9	+2.43*
*TRANSPARENT TESTA 8 (TT8)*	+2.30	+1.56
**Transport and Compartmentation Genes**		
*TRANSPARENT TESTA 12 (TT12)*	+8.08	+2.35*
*TRANSPARENT TESTA 19 (TT19)*	+13.98	+1.78**
*AUTOINHIBITED H+-ATPASE ISOFORM 10 (AHA10)*	+4.32	+1.58

Asterisks indicate P-value * <0.05, ** <0.01.

**Figure 5:**
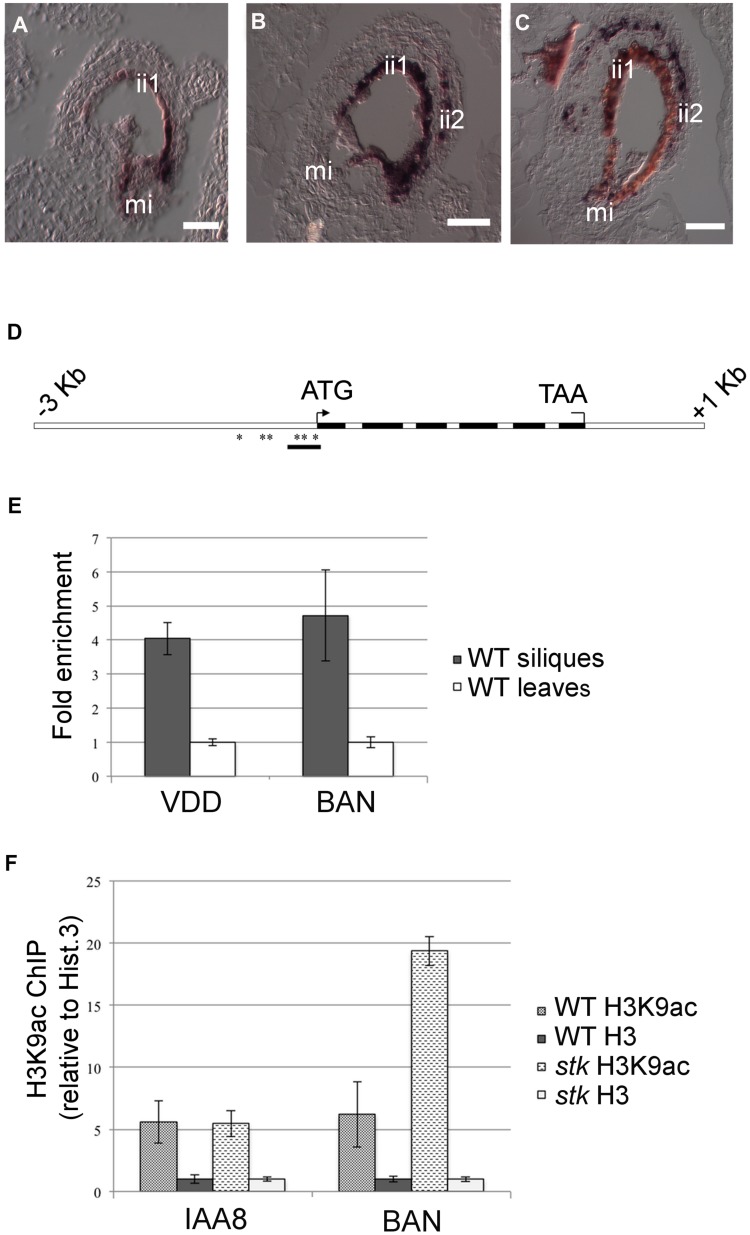
STK directly regulates *BAN* expression through the modification of chromatin state. (*A*) *In situ* hybridization experiments illustrating the expression of the *BAN* transcript: in wild-type seeds at 1 DAP, *BAN* is expressed only in the endothelium layer. (*B*) *BAN* expression in the *stk* mutant background at 1 DAP and (*C*) at 4 DAP. In the *stk* mutant background, *BAN* expression is affected: the *BAN* transcript was detected not only in the endothelium layer but also in the ii2 layer. (*D*) Schematic representation of CArG box positions indicating the regions analysed by the ChIP experiment (black bars). Black boxes: exons; white boxes: promoter, introns, 3′ and 5′ UTRs. Asterisks indicate CArG boxes. (*E*) ChIP enrichment tests by qRT-PCR show that STK binds to the selected region of *BAN*. Fold enrichment was calculated over the negative controls. Error bars represent the propagated error value using three replicates. (*F*) ChIP enrichment tests by qRT-PCR show that STK negatively correlates with the H3K9ac acetylation mark at the *BAN* translational start site. qRT-PCR quantification of *BAN* sequences in precipitated chromatin was used to infer the acetylated histone H3 and total histone H3 representation at the STK-binding site. Levels of histone modification were normalized to total histone H3. Ct values were used to calculate the IP/IN signal. ChIP enrichments are presented as the percentage (%) of bound/input signal. ChIP enrichments for H3K9ac were normalized to histone H3 density. We tested the efficiency of IP by quantifying the presence of the H3K9ac mark in IAA8 [49] which was shown to be strongly and equally expressed in both samples and yielded equal enrichment ratios. mi, micropyle; ii1, endothelium; ii2, internal layer of inner integument. Scale bars = 40 µm (*A–C*).

To investigate in which seed tissues *BAN* is expressed we performed *in situ* hybridization experiments using an antisense *BAN* probe. In wild-type seeds *BAN* was expressed only in the endothelium layer (Fig. 5A; [20]). In contrast, *BAN* expression was observed in the endothelium layer and ectopically in the ii2 layer in the *stk* mutant (Fig. 5B–C). These data confirm that STK controls the spatial and temporal expression of *BAN*.

### The SEEDSTICK-GFP reporter line reveals a dynamic fluorescence pattern in developing ovules and seed integuments

**Figure 6:**
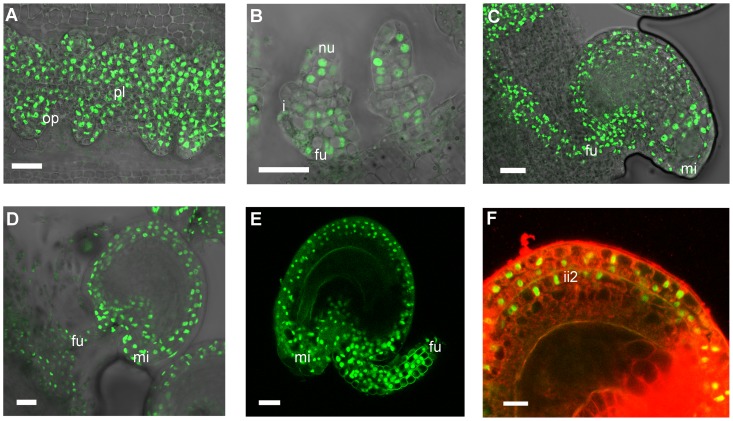
Confocal laser-scanning images of *pSTK::STK-GFP* expression patterns during ovule and seed development. (*A*) Early ovule development: STK-GFP nuclear protein is expressed in the placenta and in the ovule primordia. (*B*) When integuments arise STK-GFP signal is localized in the nucellus and in the funiculus. (*C*, *D*) Mature ovule development: GFP can be detected throughout the integuments, funiculus and the adjacent placental region. (*E*) After fertilization the STK-GFP signal is present in the outer integuments and funiculus of developing seeds. (*F*) Magnification of figure E with an overlay projection images of specific PI staining to determine the presence of the GFP signal in the integuments. Protein can be detected in the two layers of the outer integument and also in the more external layer of the inner integument. op, ovule primordia; pl, placenta; nu, nucellus; i, integuments; fu, funiculus; mi, micropyle; ii2, internal layer of inner integument. Scale bars = 50 µm (*A* and *B*), 40 µm (*C*, *D* and *E*) and 20 µm (*F*).

To investigate how STK regulates *BAN* expression we examined the expression pattern of the STK protein. We cloned the entire *STK* genomic region (a DNA fragment comprising 3.5 kb of sequence upstream of the ATG codon plus all the coding region) as a translational fusion to a GFP reporter gene, and the final *pSTK::STK-GFP* construct was introduced into the *stk* mutant background. The resulting plants produced seeds that were indistinguishable from wild-type and were able to abscise from the fruit upon maturity, demonstrating that the STK-GFP fusion protein was biologically active and able to fully complement the absence of the endogenous STK protein. STK-GFP was uniformly detected in the nuclei of the placenta and the early ovule primordia (Fig. 6A–B) which is consistent with previous data showing *STK* mRNA expression from stage 9 of flower development [7]. Later, as the developing ovules initiate the inner and outer integuments, GFP expression was found to be restricted to the nucellus and the funiculus (Fig. 6B). However, during subsequent stages the fluorescence signal appeared throughout the outer and inner integuments (Fig. 6C–D), and as development proceeded it could be seen to be present strongly in the growing funiculus and also in its contact region with the placenta. This pattern of protein localization during ovule development is consistent with previous *in situ* hybridization data [7]. During early embryogenesis (Fig. 6E) the STK-GFP protein remained extended throughout the outer integuments. Cell wall specific staining with propidium iodide (PI) was also carried out and allowed us to better define the sites of STK-GFP localization as the outer integuments and the second layer of the inner integument (Fig. 6F).

Our observations thus show that the distribution of the STK protein changes over the course of ovule and seed development. During early stages it is detected uniformly distributed in the placenta and the different cell types of the ovule primordia, whereas later it becomes restricted to the maternal seed coat and funiculus. No STK-GFP was detected in the ii1 and ii1′ layers suggesting that here STK does not repress *BAN* in these tissues. These data are consistent with the observation that STK is able to repress *BAN* expression only in the ii2 layer of the inner integument.

### STK binds to the *BAN* promoter affecting histone H3K9 acetylation levels

RNA-Seq, expression analysis and *in situ* data all indicate that *BAN* expression is regulated by STK. In order to investigate whether this regulation involved direct interaction between the STK protein and the *BAN* gene we performed a ChIP (Chromatin Immunoprecipitation) assay on wild-type inflorescences and siliques (up to 6 DAP) using an antibody specific against the STK protein. Chromatin extracted from wild-type leaves was used as a negative control since *STK* is not expressed in this tissue, and the binding of STK to the *VERDANDI* (*VDD*) promoter [43] was used as the positive control. Bearing in mind that MADS-domain proteins recognize and bind CArG boxes [44], the *BAN* gene genomic sequences comprising the 3 Kb upstream of the ATG start codon, the structural gene and 1 kb downstream of the STOP codon were analysed for the presence of such consensus motifs (allowing up to one base mismatch; Fig. 5D). Six CArG boxes were found in the region of the *BAN* promoter sequence [2] extending up to 355 bp upstream from the ATG. We detected significant enrichment for the region immediately upstream of the *BAN* translational start site (primer set spanning positions −140 to +1, including three CArG boxes (Fig. 5E)) thus indicating the presence of STK bound to this region.

To investigate a possible mechanism of regulation of the expression of *BAN* by STK, we examined epigenetic marks at the *BAN* locus. Modifications such as the hyperacetylation of histones H3 and H4 have diverse impacts on gene transcriptional activity and chromatin organization [45]. H3K9ac is one of the most well-characterized epigenetic marks associated with active transcription and has been shown to influence numerous developmental and biological processes in higher plants [46]–[48]. To address the question of whether the differential expression of *BAN* observed between *stk* and wild-type tissues correlates with alterations in this epigenetic marker we analyzed wild-type and *stk* mutant siliques at 3–4 DAP for H3K9 acetylation at the *BAN* locus. ChIP experiments were performed using an antibody specific to H3K9ac and were analyzed by qRT-PCR (Fig. 5F). *IAA8* was used as a reference as it carries the H3K9ac mark and is equally expressed in both wild-type and *stk* mutant plants [49]. Interestingly, in wild-type material we found considerable enrichment of DNA sequences corresponding to the region around the translational start site within which STK binding sites are located. When we assayed the same region in the *stk* mutant we observed a dramatic increase in enrichment compared to wild type. These results are consistent with the presence of elevated levels of H3K9ac (compared to H3) in the region of the wild-type *BAN* promoter where STK interacts (transcriptionally active chromatin), and in addition evidences a very considerable enrichment of H3K9ac in this region in the *stk* mutant which correlates with the increased transcriptional activity of the *BAN* gene observed in the mutant background.

### STK controls *BAN* transcriptional regulators

**Figure 7:**
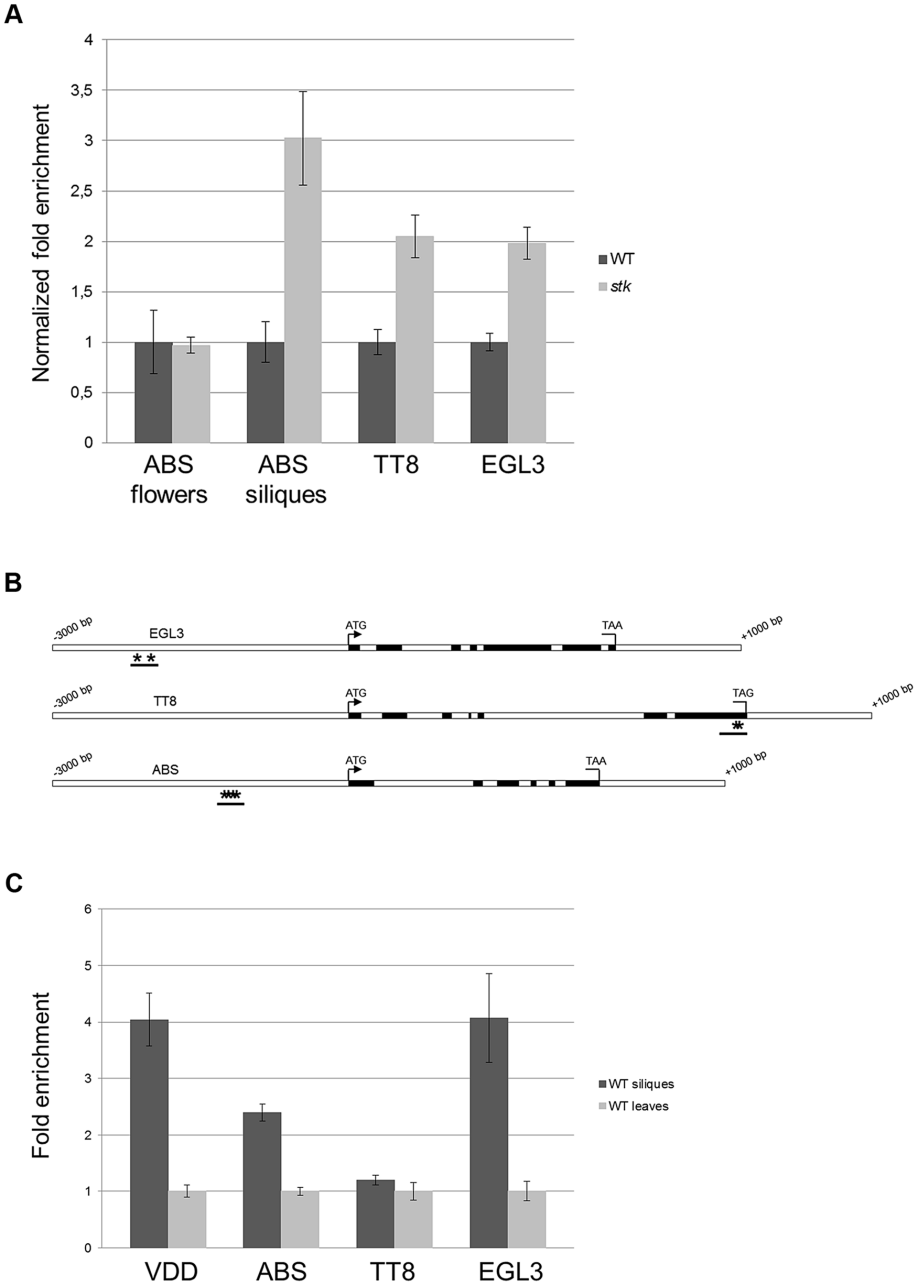
*BAN* regulators are direct targets of STK. (*A*) qRT-PCR performed on cDNA obtained from siliques from 3 to 4 DAP and from unpollinated flowers for *ABS*. Relative mRNA levels indicate that the expression of all the genes is up-regulated in the absence of the STK protein at 3–4 DAP; these differences are statistically significant as determined by Statistical Student's *t* test (*P*<0.01). The expression level of *ABS* is not affected in unpollinated flowers in the absence of the STK protein. Error bars represent the propagated error value using three replicates. (*B*) Schematic representation of CArG box positions. Schematic diagrams of EGL3, ABS and TT8 loci indicating the regions analysed by the ChIP experiment (black bars). Black boxes: exons; white boxes: promoters, introns, 3′ and 5′ UTRs. Asterisks indicate CArG boxes. (*C*) ChIP enrichment tests by qRT-PCR show that STK binds to the selected regions of *ABS* and *EGL3*. Fold enrichment was calculated over the negative controls. Error bars represent the propagated error value using three replicates.

Our data suggest that STK might regulate *BAN* expression directly binding to its promoter. Previous studies have identified other key regulators of BAN expression, including ABS, TT8 and ENHANCER OF GLABRA3 (EGL3). Whereas TT8 and EGL3 act redundantly in a protein complex that promotes the expression of *BAN*, ABS is necessary for PA biosynthesis and normal endothelium cell morphology [9], [28], [41], [42], [50]. This raised the question as to whether STK might act as master regulator also controlling the genes encoding transcription factors that regulate *BAN*. We therefore investigated the expression of *ABS*, *EGL3* and *TT8* in both wild-type and *stk* mutant siliques (3–4 DAP) by qRT-PCR, and also analyzed the expression of *ABS* in developing flowers in order to study the relationship between *STK* and *ABS* in their roles in endothelium formation [9]. This experiment revealed up-regulation of *TT8*, *EGL3* and *ABS* in *stk* mutant siliques and hence that STK represses these genes in the wild type at this specific stage of development (Fig. 7A); that the level of *ABS* in un-pollinated inflorescences was unaffected indicates that STK does not play a role in regulating the expression of *ABS* prior to pollination. These data therefore suggest that STK regulates these genes during a narrow stage of development, at 3–4 DAP.

In order to clarify whether *STK* directly controls the expression of these regulatory genes, we performed a ChIP assay. Bioinformatics analysis of the gene loci revealed the presence of two CArG boxes in the putative *EGL3* promoter region at −2213 and −2177 bp; three CArG boxes in the putative *ABS* promoter region between −1692 and −1599 bp, and the *TT8* gene presented two CArG boxes in the last exon at +3805 and +3815 bp from the ATG (Fig. 7B). The ChIP assays showed that STK indeed binds to the CArG-containing regions of the *EGL3* and *ABS* genes but not to the selected region of *TT8* (Fig. 7C). This analysis suggests that the control of PA biosynthesis by STK might occur via two mechanisms: a direct interaction with the *BAN* locus, and in addition by direct and indirect regulation of the expression of genes that encode transcriptional regulators of *BAN*.

## Discussion

Seed development is a highly complex process that includes the formation of the zygote, storage tissues and a protective seed coat. Differentiation of the various structures is evidenced at the morphological level but is also reflected by the spatial distribution of metabolites. *STK* is involved in seed development in several ways, for example *stk* mutant seeds are smaller than those of wild type and do not detach from the mother plant. Furthermore, combining the *stk* mutant with the *arabidopsis b-sister* (*abs*) mutant resulted in ovules that failed to develop the endothelium layer that forms the innermost component of the seed coat [9]. To gain a deeper insight into the role of *STK* during ovule and seed development we performed RNA-Seq on material extracted from wild-type and *stk* mutant inflorescences and seeds. The resulting transcriptomic analysis yielded a list of 246 genes identified as being differentially expressed between *stk* and wild type. The majority of these (156 genes) were up-regulated in the *stk* background whilst 90 were down-regulated, which suggests that *STK* may act primarily as a repressor. Of all the deregulated transcripts, we found 18 that were expressed in the *stk* mutant but not in wild type, and 11 that were specifically expressed in the wild type but not in *stk*. GO analysis of the genes that were down-regulated in the *stk* mutant background revealed that they encoded proteins involved in DNA binding, including group II WRKY transcription factors (TFs) such as WRKY39 [51].

The importance of TTG2 (WRKY group I) as a regulator of the LBG biosynthetic pathway has been described previously [16]. Our RNA-Seq data revealed that expression levels of TTG2 are unaltered between wild type and *stk*. WRKY TFs are global regulators acting at various levels, including the direct modulation of immediately downstream target genes, but they also appear to interact with key chromatin-remodelling factors [52]. The latter could be of special interest since we found that the STK-dependent *BAN* regulatory mechanism involves a chromatin remodelling activity which may imply the action of downstream STK targets acting as remodelers that have yet to be defined. In this regard, the nucleosome assembly/disassembly protein NAP1-RELATED PROTEIN 1 (NRP1) which interacts with chromatin remodelling factors [53] was also found in this list. In addition we identified NAC transcription factor-like 9 (NTL9), a calmodulin-regulated NAC transcriptional repressor in Arabidopsis [54], and SQUAMOSA PROMOTER BINDING PROTEIN-LIKE 2 (SPL2), a member of the SQUAMOSA PROMOTER BINDING PROTEIN (SBP)-box family of transcription factors [55]. Future analysis will be directed to determine the possible mechanism(s) of action of STK.

GO analysis of genes that are up-regulated in the *stk* mutant showed an abundance of transcripts involved in secondary metabolic processes, including those involved in flavonoid and phenylpropanoid biosynthesis. We hypothesize that STK may act on the PA pathway at two different levels: i) impacting directly on the elements comprising the enzymatic pathway of PA biosynthesis, and/or ii) influencing key regulatory elements. In the first case, all the genes of the Late Biosynthetic step were found to be up-regulated in the *stk* mutant. However, no changes were found for the group of genes involved in the metabolic steps corresponding to early synthesis. This may indicate that STK regulation does not involve major alterations at the level of production of dihydroflavonols, the precursors to PAs, but impact instead on the synthesis of anthocyandins and proanthocyanidins. We also found that two out of the three genes involved in the pathway controlling transport and compartmentation were up-regulated: TT12 and TT19. Since anthocyanins are transported from the cytosol to the vacuole as part of the PA biosynthetic pathway, it may be reasonable to expect coordinated regulation of transporter-mediated and vesicle-mediated mechanisms by STK. As commented above, STK may also act on key regulatory elements. In this regard we observed a strong influence on the expression levels of the members of the bHLH transcription factor family (*TT8* and *EGL3*), the Zinc-Finger transcription factor (*TT1*) and the R2R3 MYB domain putative transcription factor (*TT2*) in the *stk* mutant background. These regulatory elements together with other key MADS-domain transcription factors like *ABS* were all found to be up-regulated.

Based on observation from the RNA-Seq data we investigated the role of STK in PA biosynthesis in more detail. Histological analysis revealed that PAs accumulated ectopically in the outer-layer of the inner integument in *stk* mutant seeds, and analysis by LC-MS analysis demonstrated that the level of soluble epicatechin monomers was greater in the *stk* mutant in both immature and mature seeds. The latter data confirm the morphological analysis (Fig. 2) and support the idea that STK is involved in PA accumulation and in particular the accumulation of epicatechin monomer.

Regulation of the production of flavonoids and proanthocyanidins has been extensively studied (for review see [16]). Several transcription factors are known to be involved in the regulation of *BAN* expression, and in particular a R2R3-MYB/bHLH/WDR complex is responsible for *BAN* activation in the endothelium [25], [40]–[42]. Not all the members of this complex exhibit the same hierarchy, for instance it has been suggested that TT8 is necessary for activation of the LBGs whereas a predominant role of TT2 controlling via a positive feedback loop is required to maintain the transcript levels of both *TT8* and *BAN* [2], [25], [56]. Interestingly our RNA-Seq dataset highlighted a repressive role for STK on the regulation of *TT2*. Another gene required for the activation of *BAN* in the endothelium is *ABS*. This gene is a member of the MADS-domain gene family and to date it is the only MADS-domain gene identified to be involved in the control of PA accumulation. *abs* mutants show significant reductions in epicatechin and procyanidins accumulation [10]; moreover, in the *abs* background both GUS reporter gene expression driven off the *BAN* promoter and qPCR analysis of *BAN* expression itself showed significant down-regulation [28], [57]. In the complex regulatory network that governs PA accumulation in the endothelium layer, STK plays a key role controlling *BAN* expression, and our results suggest that this occurs through the binding of STK to the *BAN* regulatory region. Recently, Dean and collaborators performed genome-wide expression profiling using microarrays to identify those genes differentially expressed in the wild-type and *abs* mutant seed coats [57]. This showed that *STK* expression is unaffected in the *abs* mutant at 3 DAP but is up-regulated at 7 DAP. Furthermore, Nesi and colleagues demonstrated that ectopic expression of *ABS* causes altered PA accumulation in a manner very similar to that which we have observed in the *stk* mutant, since these plants present PAs not only in the endothelium but also in the more external layer of the inner integuments [28]. Integration of previous data with our results indicates that STK is a master regulator of inner seed coat differentiation. Whilst several transcription factors involved in seed coat determination or secondary metabolite accumulation have been already characterized [58], no gene connecting these processes has been described previously. In our study we provide evidence of a role for chromatin modification, specifically H3K9 acetylation, in the transcriptional regulation of *BAN*. We have shown that the region of the *BAN* promoter proximal to the translational start site is heavily covered by this epigenetic marker of transcriptional activity. H3K9ac enrichment is greater when *BAN* is ectopically expressed due to the lack of STK protein. This suggests that STK somehow represses the activity of histone deacetylases (HDACs) at the *BAN* locus in cells of the outer layer of the inner integument. It will be interesting to determine whether STK is able to recruit a chromatin remodelling partner, yet to be identified, that would form part of a hypothetical STK complex. It is known that histone acetyltransferases (HATs) and HDACs participate in the genome-wide turnover of acetyl groups, and that besides histones some also modify other factors. Future progress will therefore be focused on determining their availability for interaction with specific transcription factors like STK and other protein complex partners.

PA biosynthesis and its spatial accumulation are under the control of a complex regulatory network. *BAN* is one of the key genes of the LBG group and its expression falls under the influence of several transcription factors, including ABS, EGL3 and TT8. Our work adds STK to this list. Our data also suggest STK to be a master regulator of PA biosynthesis and accumulation since we observed that it also controls the expression of *ABS* and *EGL3*. This shows that STK not only acts as a regulator of ovule identity but also orchestrates important aspects of seed development, adding new evidence for the importance of MADS-domain genes in the control of plant developmental processes.

The role of *STK* in the regulation of epicatechin accumulation could have relevance for certain agricultural applications: PAs are important in several aspects of plant protection and their significance in the flavour and astringency of foods and beverages is already known (for review see [59]). Indeed, it has been demonstrated that avocado fruits that contain higher levels of epicatechin exhibit stronger resistance to fungal attack [60]. In this regard it will be interesting to study the fungal resistance of *stk* mutant seeds since regulation of STK levels might provide a tool to make plant seeds more resistant to fungi.

## Materials and Methods

### Plant material and growth conditions

*Arabidopsis thaliana* wild-type (ecotype Columbia) and *stk* mutant plants were grown at 22°C under short-day (8 h light/16 h dark) or long-day (16 h light/8 h dark) conditions. The Arabidopsis *stk* mutant was kindly provided by M. Yanofsky [7]. The *stk-2* allele contains a 74 nucleotide insertion near the splice site of the third intron.

### PCR-based genotyping

Identification of *STK* wild-type and mutant alleles was performed by PCR analysis using oligonucleotides AtP_204 (5′-GCTTGTTCTGATAGCACCAACACTAGCA-3′) and AtP_561 (5′-GGAACTCAAAGAGTCTCCCATCAG-3′). The mutant allele yields a 399 bp DNA fragment whilst the wild-type allele produces a 325 bp fragment.

### RNA extraction, cDNA library preparation, and sequencing for RNA-Seq

Total RNA was extracted from two biological replicates (1 gr) from both wild-type and *stk* mutant inflorescences and siliques until 5 DAP using the Qiagen ‘RNeasy miniKit’ according to the manufacturer's instructions. DNA contamination was removed using PROMEGA RQ1 RNase-Free DNase according to the manufacturer's instructions. RNA quality and integrity were analyzed by gel electrophoresis and validated on a Bioanalyzer 2100 (Aligent, Santa Clara, CA); RNA Integrity Number (RIN) values were greater than 7 for all samples. In order to confirm that the *stk* mutant was a knock-out line, *STK* expression was checked by qRT-PCR with primers RT_780 (5′-TGCGATGCAGAAGTTGCGCTC-3′) and RT_781 (5′-AGTACGCGGCATTGATTTCTTG-3′). Sequencing libraries were prepared according to the manufacturer's instructions using the TruSeq RNA Sample Prep kit (Illumina Inc.) and sequenced on an Illumina HiSeq2000 (50 bp single-read). The processing of fluorescent images into sequences, base-calling and quality value calculations were performed using the Illumina data processing pipeline (version 1.8). Raw reads were filtered to obtain high-quality reads by removing low-quality reads containing more than 30% bases with Q<20. Finally, quality control of the raw sequence data was performed using FastQC (http://www.bioinformatics.babraham.ac.uk/projects/fastqc/).

### Mapping of short reads, quality analysis and assessment of gene expression analysis for RNA-Seq

Evaluation and treatment of raw data was performed on the commercially available CLC Genomics Workbench v.4.7.1 (http://www.clcbio.com/genomics/). After trimming, the resulting high-quality reads were mapped onto the Arabidopsis genome (TAIR10). Approximately 25M reads of each sample that mapped with ≤2 mismatches were used for further analyses. The read number of each gene model was computed based on the coordinates of the mapped reads. A read was counted if any portion of that read's coordinates were included within a gene model. As CLC Genomics Workbench v.4.7.1 distributes multireads at similar loci in proportion to the number of unique reads recorded and normalized by transcript length, we included both unique reads and reads that occur up to 10 times in the analysis to avoid undercount for genes that have closely related paralogs [36]. Gene expression values were based on reads per kilobase of exon model per million mapped read (RPKM) values [36]. The fold change and differential expression values between wild type and the *stk* mutant was calculated in terms of RPKM of the corresponding transcripts. Statistical analysis of biological replicates was assessed using heat map visualization of Euclidean distances. This clustered the biological replicates in the wild-type and mutant groups as expected. We further checked whether the overall variability of the samples reflected their grouping by performing Principal Component Analysis (PCA). This confirmed that the replicates were relatively homogenous and distinguishable from the samples of the other group. Finally, the overall distribution of expression values between the different samples confirmed that none of the samples stood out from the rest. To obtain statistical confirmation of the differences in gene expression, P and FDR values were computed using Baggerley's test on expression proportions. We applied a threshold value of *P* = 0,05 to ensure that differential gene expression was maintained at a significant level (5%) for the individual statistical tests. Transcripts that exhibited an estimated absolute Fold Change ≥2 (*i.e.* 2 mapped reads per kilobase of mRNA) were determined to be significantly differentially expressed. To gain insight into the biological processes associated with the regulated genes, we determined which GO annotation terms were over-represented, in both the up-regulated (S1 Table) and down-regulated (S2 Table) lists. Gene set enrichment analysis was performed with the agriGO database [38] using the Singular Enrichment Analysis (SEA). All RNA-seq files are available from the NCBI GEO database (accession number GSE59637).

### Histological analysis

For the morphological analysis of integuments, *Arabidopsis thaliana* wild-type (ecotype Columbia) and *stk* mutant plants were fixed for Technovit 7100 embedding (Heraeus Kulzer) following the manufacturer's instructions. Sections of plant tissue (0.8 µm) were stained in 0.5% (w/v) toluidine blue O. Samples were observed using a Zeiss Axiophot D1 microscope (http://zeiss.com/) equipped with differential interface contrast (DIC) optics. Images were recorded with an Axiocam MRc5 camera (Zeiss) using the Axiovision program (version 4.1). The whole-mount vanillin assay for PA detection was performed as described previously [61]. Vanillin (vanilaldehyde) condenses specifically with PAs and flavan-3-ol precursors to yield a bright-red product under acidic conditions. Microscopic observations were performed as detailed above.

### Preparation of extracts

Three biological replicates of immature and mature seeds (30 mg) were frozen in liquid nitrogen and ground to a fine powder using an analytical mill (IKA; A11 basic). The soluble PA fraction was extracted with 75% methanol∶water (v/v) containing 0.1% formic acid. The mixture was vortexed, sonicated for 30 min at room temperature, vortexed again and then centrifuged (13000 rpm, 10 min). Soluble PAs (contained in the supernatant) were analyzed by LC-MS. The pellet was washed with 50% methanol∶water, centrifuged (13000 rpm, 10 min), washed again with 100% methanol and centrifuged (13000 rpm, 10 min). Samples were dried in a speed vacuum for 1 hour at 30°C and then hydrolyzed with NaOH (2N) for 15 min at 60°C. The mixture was vortexed and HCl (4N) was added. To remove lipids hexane was added to the mixture and after centrifugation (13000 rpm, 10 min) the upper phase was removed. The insoluble PAs were extracted three times with ethyl acetate, and the three extractions were combined and dried in a speed vacuum for 1 hour at 30°C. The pellet was dissolved in acetone∶water∶acetic acid (70∶29.5∶0.5 v/v/v), vortexed, sonicated for 15 min and centrifuged (13000 rpm, 10 min). This PA fraction was analyzed by LC-MS.

### Targeted profiling of proanthocyanidins

The profiling of PAs in extracts of seeds was performed by MS analysis using the UPLC-qTOF instrument (Waters High Definition MS System; Synapt) with the UPLC column connected online to a photodiode array detector (Waters, Acquity) and then to the MS detector equipped with an electrospray probe. The separation of metabolites and detection of the eluted compound masses was performed as previously described [62]–[66].

### Construction of binary vectors and plant transformation

For the construction of *pSTK::STK-GFP*, DNA fragments containing the 3,5 kb promoter region and the complete *STK* genomic region without the stop codon were amplified from wild-type genomic DNA and cloned into a pGreen II binary vector containing a *GFP* reporter gene cassette. For amplification we used the following primers containing attB1 and attB2 recombination sequences: AtP_3066 5′-GGGGACAAGTTTGTACAAAAAAGCAGGCTCCAACCAATATCACACCCTAAATAC-3′ and AtP_3067 5′-GGGGACCACTTTGTACAAGAAAGCTGGGTCGTCCGAGATGAAGAATTTTCTTGTC-3′.

The resulting binary vectors were transformed into *Agrobacterium tumefaciens* by electroporation prior to stable transformation of plants using the floral dip method. Transformant lines were obtained using BASTA as a selection agent. Resistant transgenic plants showing strong GFP fluorescence were genotyped and those that complemented the *stk* phenotype were selected. Protein expression patterns were analyzed by Confocal Laser Scanning Microscopy (CLSM). Fresh material was collected, mounted in 10 mg/ml of propidium iodide (Sigma P-4170) in water and immediately analyzed. CLSM analysis was performed using a Leica TCS SPE with a 488 nm argon laser line for excitation of GFP fluorescence; emissions were detected between 505 and 580 nm. For PI fluorescence a 543 nm laser line was used and emissions were detected between 600 and 640 nm. Confocal scans were performed with the pinhole at 1 airy unit. Images were collected in the multi-channel mode and the overlay images were generated using the Leica analysis software LAS AF 2.2.0.

### Expression analysis by *in situ* hybridization

DIG-labelled RNA probes for detection and hybridization of *BAN* were prepared as previously described [67]. Sections of plant tissue were hybridized with digoxigenin-labelled *BAN* antisense probe amplified using primers AtP_4331 (5′-CGAGTAGC TTATCTCTCTCG -3′) and AtP_4332 (5′- TCAATCCTTTTGACTCGAAG -3′).

### ChIP and qRT-PCR analysis

The genomic regions located 3 kb upstream of the ATG, 1 kb downstream of the stop codon, and in the exons and introns of the selected genes were analyzed to identify CArG box sequences with up to one base mismatch. ChIP experiments were performed in a modified version of a previously reported protocol [68]. The qRT-PCR assay was conducted in triplicate on three different biological replicates, with three technical replicates for each sample, and was performed in a Bio-Rad iCycler iQ optical system (software version 3.0a). ChIP efficiency was determined using the third CArG box of the *VDD* gene as a positive control [43]. Fold enrichment was calculated using the formulae of a previously reported protocol [43].

For ChIP-based analysis of histone modifications, the following antibodies were used for immunoprecipitation: rabbit anti-histone H3 (Sigma-Aldrich H0164) and rabbit anti-H3 acetyl K9 (Upstate 07-352) and were handled in parallel to samples lacking antibody. qRT-PCRs were performed on input and immunoprecipitated samples and % of input was calculated. The signal obtained after precipitation with anti-H3K9ac antibody (as indicated in the figure) was normalized to the signal obtained by precipitation with an antibody to an invariant domain of histone H3. For H3K9ac analyses, *IAA8* (*At2g22670*) was used as a reference as it carries the H3K9ac mark and is equally expressed in both samples [49]. Relative enrichment of *Mu-like* transposons was included as negative control. Sequences of oligonucleotides used for ChIP analyses are listed in S5 Table.

### Expression analysis

qRT-PCR experiments were performed on cDNA obtained from siliques from 3 to 4 DAP and unpollinated flowers. Total RNA was extracted using the LiCl method [69]. DNA contamination was removed using the Ambion TURBO DNA-free DNase kit according to the manufacturer's instructions. The treated RNA was reverse transcribed using the ImProm-II reverse transcription system (Promega). cDNAs were used as templates in the qRT-PCR reactions containing the iQ SYBR Green Supermix (Bio-Rad). The qRT-PCR assay was conducted in triplicate on three different biological replicates, with three technical replicates for each sample, and was performed in a Bio-Rad iCycler iQ Optical System (software version 3.0a). Relative transcript enrichment of genes of interest was calculated normalizing the amount of mRNA against different endogenous control fragments (*UBQ*, *ACT*, *PPa2* and *SAND* [70]). The difference between the cycle threshold (Ct) of the gene and that of the reference gene (ΔCt = CtGENE – CtREFERENCE) was used to obtain the normalized expression of that gene, which corresponds to 2^−ΔΔCt^. The primers used for this analysis are listed in S5 Table.

### Accession number

Annotated sequences used in this article can be found in the GenBank/EMBL data libraries under the following accession number: *SEEDSTICK*, *At4g09960*; *BANYULS*, *At1g61720*.

## Supporting Information

S1 Figure Insoluble PAs analysed by LC-MS. No differences were detected in the metabolic profiles of insoluble PAs between mature wild-type (black bars) and *stk* mutant seeds (grey bars). Error bars represent SD of three independent measurements.
(TIF)

S2 Figure *BAN* expression is up-regulated in the *stk* mutant. qRT-PCR performed on cDNA obtained from siliques from 0 to 6 DAP. The relative mRNA levels confirmed the result obtained by the RNA-Seq experiment indicating that the expression of BAN is up-regulated in the absence of the STK protein. Error bars represent the propagated error value using three replicates.
(TIF)

S1 Table List of 156 genes up-regulated in the *stk* mutant background according to the criteria described in the text.
(XLS)

S2 Table List of 90 genes down-regulated in the *stk* mutant background according to the criteria described in the text.
(XLS)

S3 Table Gene ontology analysis. Singular enrichment analysis of GO annotation terms over-represented in the list of genes up- and down-regulated in the Arabidopsis *stk* mutant with respect to the wild-type.
(DOCX)

S4 Table List of the annotated metabolites shown in Fig. 3.
(DOCX)

S5 Table List of primer pairs used in the ChIP and expression analysis experiments.
(DOC)
