# Supplementary figures and images for: SEEDSTICK is a Master Regulator of Development and Metabolism in the Arabidopsis Seed Coat
Source: PLoS Genet. 2014 Dec 18;10(12):e1004856. doi: 10.1371/journal.pgen.1004856 (PMC4270456; doi:10.1371/journal.pgen.1004856)

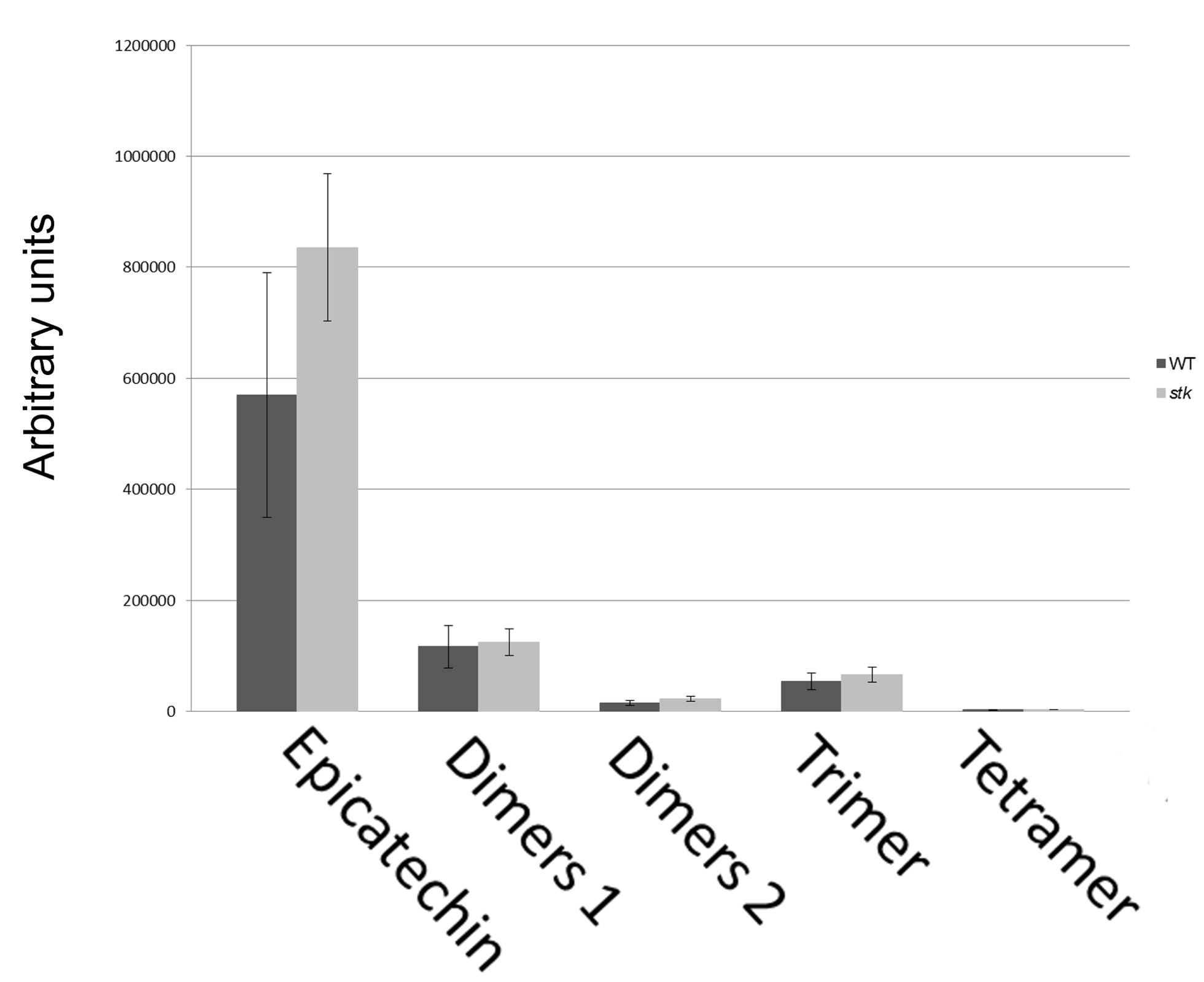

Supplement: S1 Figure — Insoluble PAs analysed by LC-MS. No differences were detected in the metabolic profiles of insoluble PAs between mature wild-type (black bars) and stk mutant seeds (grey bars). Error bars represent SD of three independent measurements. (TIF) [file pgen.1004856.s001.tif]

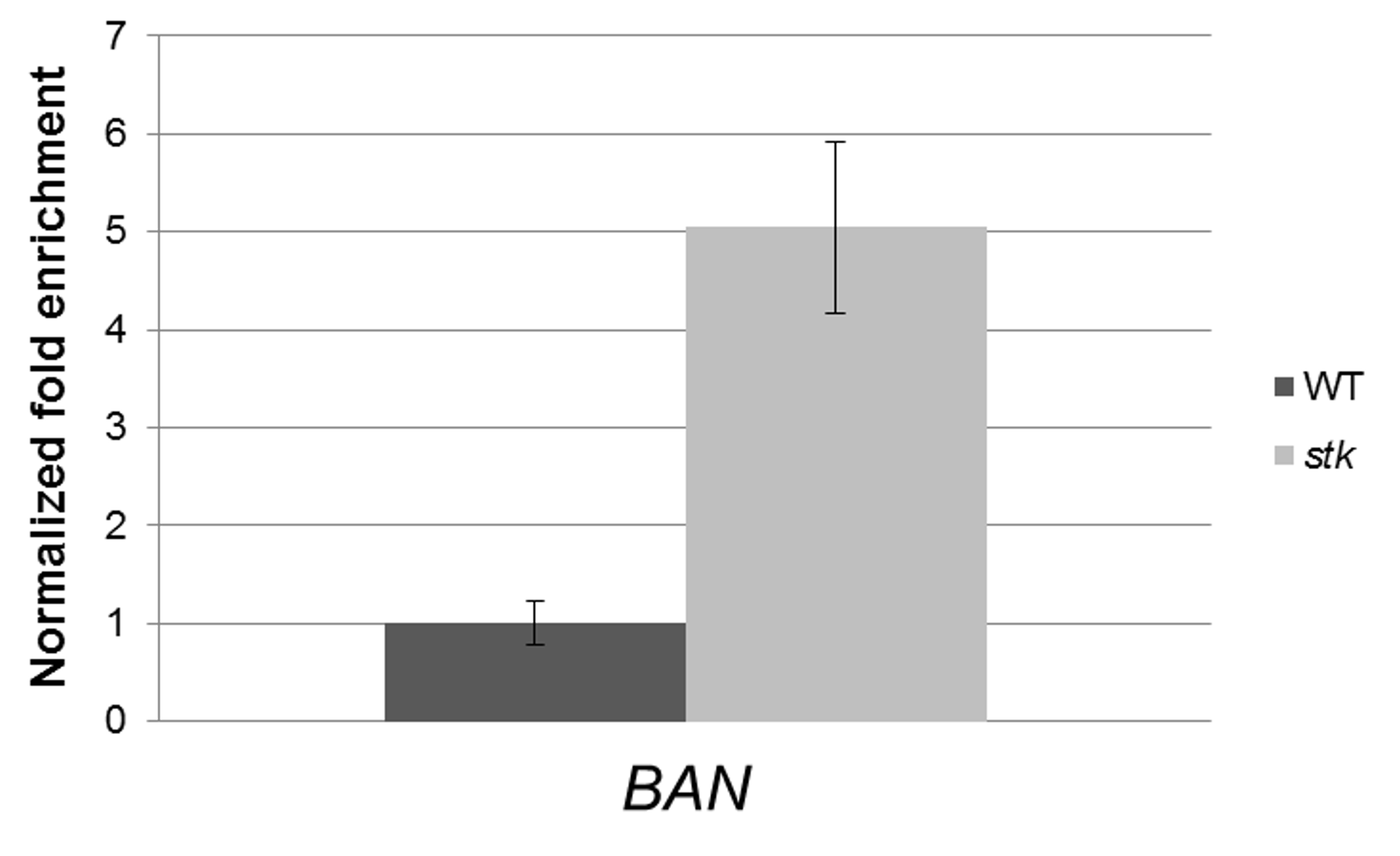

Supplement: S2 Figure — BAN expression is up-regulated in the stk mutant. qRT-PCR performed on cDNA obtained from siliques from 0 to 6 DAP. The relative mRNA levels confirmed the result obtained by the RNA-Seq experiment indicating that the expression of BAN is up-regulated in the absence of the STK protein. Error bars represent the propagated error value using three replicates. (TIF) [file pgen.1004856.s002.tif]
