# Supplementary material for: SEEDSTICK is a Master Regulator of Development and Metabolism in the Arabidopsis Seed Coat
Source: PLoS Genet. 2014 Dec 18;10(12):e1004856. doi: 10.1371/journal.pgen.1004856 (PMC4270456; doi:10.1371/journal.pgen.1004856)
Supplement: S3 Table — Gene ontology analysis. Singular enrichment analysis of GO annotation terms over-represented in the list of genes up- and down-regulated in the Arabidopsis stk mutant with respect to the wild-type. (DOCX) [file pgen.1004856.s005.docx]

**Table S3. Gene ontology analysis.**

Singular enrichment analysis of GO annotation terms over-represented in the list of genes up- and down-regulated in the Arabidopsis *stk* mutant with respect to the wild-type (with a p-value <0,005).

|  | **Go term** | **p-value** |
| --- | --- | --- |
| **A) UP-REGULATED** |  |  |
| **CELLULAR COMPONENT** | | |
|  | endomembrane system | 2.9 E-10 |
|  | cell part | 6.9 E-9 |
|  | cell | 6.9 E-9 |
| **MOLECULAR FUNCTION** | | |
|  | pectinesterase activity | 0.00032 |
|  | lipid binding | 0.00035 |
|  | enzyme inhibitor activity | 0.00071 |
|  | enzyme regulator activity | 0.00073 |
| **BIOLOGICAL PROCESSES** | | |
|  | lipid localization | 3.10 E-11 |
|  | secondary metabolic process | 5.10 E-06 |
|  | phenylpropanoid metabolic process | 8.30 E-06 |
|  | flavonoid biosynthetic process | 1.10 E-05 |
|  | flavonoid metabolic process | 2.00 E-05 |
|  | phenylpropanoid biosynthetic process | 2.70 E-05 |
|  | cellular amino acid derivative metabolic process | 4.50 E-05 |
|  | cellular amino acid derivative biosynthetic process | 4.90 E-05 |
|  | aromatic compound biosynthetic process | 5.40 E-05 |
|  | lipid transport | 5.80 E-05 |
|  | cellular aromatic compound metabolic process | 0.00022 |
|  | cellular amino acid and derivative metabolic process | 0.00043 |
|  | macromolecule localization | 0.00057 |
|  | response to abscisic acid stimulus | 0.00086 |
|  | response to endogenous stimulus | 0.0012 |
|  | response to hormone stimulus | 0.002 |
| **B) DOWN-REGULATED** | | |
| **MOLECULAR FUNCTION** |  |  |
|  | DNA binding | 3.90E-05 |
