# Supplementary material for: SEEDSTICK is a Master Regulator of Development and Metabolism in the Arabidopsis Seed Coat
Source: PLoS Genet. 2014 Dec 18;10(12):e1004856. doi: 10.1371/journal.pgen.1004856 (PMC4270456; doi:10.1371/journal.pgen.1004856)
Supplement: S4 Table — List of the annotated metabolites shown in Fig. 3. (DOCX) [file pgen.1004856.s006.docx]

**Table S4. List of the annotated metabolites shown in Figure 3.**

| **Retention time (min)** | **Mass (m/z)** | **Polarity** | **Annotation** |
| --- | --- | --- | --- |
| 6.83 | 289.07 | Negative | (Epi)Catechin |
| 6.1 | 577.13 | Negative | Dimer1 |
| 10.65 | 577.13 | Negative | Dimer2 |
| 7.74 | 865.2 | Negative | Trimer |
| 8.24 | 1153.26 | Negative | Tetramer |
| 8.52 | 720.15 | Negative | Pentamer |
| 8.96 | 864.19 | Negative | Hexamer |
| 9.36 | 1008.71 | Negative | Heptamer |
| 9.71 | 1152.75 | Negative | Octamer |
| 10.1 | 1296.79 | Negative | Nonamer |
