# Supplementary material for: SEEDSTICK is a Master Regulator of Development and Metabolism in the Arabidopsis Seed Coat
Source: PLoS Genet. 2014 Dec 18;10(12):e1004856. doi: 10.1371/journal.pgen.1004856 (PMC4270456; doi:10.1371/journal.pgen.1004856)
Supplement: S5 Table — List of primer pairs used in the ChIP and expression analysis experiments. (DOC) [file pgen.1004856.s007.doc]

**Table S5. List of primer pairs used in the ChIP and expression analysis experiments.**

| **Primers for ChIP experiment** | |
| --- | --- |
| *ACT7* | 5’-CGTTTCGCTTTCCTTAGTGTTAGCT-3’  5’-AGCGAACGGATCTAGAGACTCACCTTG-3’ |
| *VDD* | 5’-GGAAATATGACGCTTGTCTTTTTAG-3’  5’-CAGAAACAGCAATATGCTCGTG-3’ |
| *BAN* | 5’-GATCACGTGCTTACCTTC-3’  5’-TGTGTGTAAGAGTCTGGT-3’ |
| *ABS* | 5’-GAAGTTTAGACCCGTTCAAG-3’  5’-ATGCTGAAACTCGAACACTG-3’ |
| *EGL3* | 5’-TCCGTGCAATTAATGCGAGC-3’  5’-CTAACGCCGCAGATGATGATG-3’ |
| *TT8* | 5’-AAAGATAAGAGGCTACCGCG-3’  5’-ATTCTCAAGCTCATGGACCC-3’ |
| *IAA8* | 5’-GAACGTAACTACTTGGGTCT-3’  5’-GTCTTTAGAAGGTAGCAAC-3’ |
| *MU-like* | 5’-AATTCCGCCTTCGAACAACTTCTCG-3’  5’-GGCAACCCTTGCTGTTGCATTTG-3’ |
| **Primers for expression analysis experiment** | |
| *ACT8* | 5’-CTCAGGTATTGCAGACCGTATGAG-3’  5’-CTGGACCTGCTTCATCATACTCTG-3’ |
| *UBQ* | 5’-CTGTTCACGGAACCCAATTC-3’  5’-GGAAAAAGGTCTGACCGACA-3’ |
| *SAND* | 5’-CAGACAAGGCGATGGCGATA-3’  5’-GCTTTCTCTCAAGGGTTTCTGGGT-3’ |
| *PP2a* | 5’-CAGCAACGAATTGTGTTTGG-3’  5’-AAATACGCCCAACGAACAAA-3’ |
| *BAN* | 5’- ATCTTCCATGTCGCAACTCC -3’  5’- CACACGCTTGACTGATTTCG -3’ |
| *ABS* | 5’-GCAGCAACAGTTGGAGAATC-3’  5’-TCCCAGCTTGTTGAAACTCC-3’ |
| *TT8* | 5’-ATGAAGAAGCCGAAGACGAA-3’  5’-CTTGTGGGGTGTGACATGAG-3’ |
| *EGL3* | 5’-AACCAGGAGTGTTGGAGTGG-3’  5’-CGGAAGCTCGTCTAGTGACC-3’ |
